# Peripapillary and macular choroidal area in patients with normal-tension glaucoma

**DOI:** 10.1371/journal.pone.0204183

**Published:** 2018-09-13

**Authors:** Hirokazu Kojima, Kazuyuki Hirooka, Eri Nitta, Shozo Sonoda, Taiji Sakamoto

**Affiliations:** 1 Department of Ophthalmology, Kagawa University Faculty of Medicine, Miki, Kagawa, Japan; 2 Department of Ophthalmology, Kagoshima University Graduate School of Medical and Dental Sciences, Kagoshima, Japan; Massachusetts Eye & Ear Infirmary, Harvard Medical School, UNITED STATES

## Abstract

**Purpose:**

To evaluate normal and normal-tension glaucoma (NTG) eyes for differences in peripapillary and macular choroidal area measurements.

**Methods:**

This cross-sectional comparative study enrolled 52 normal subjects and 35 NTG patients. Peripapillary and macular choroidal images were recorded by enhanced depth imaging optical coherence tomography (EDI-OCT), with conversion of the luminal and interstitial areas to binary images performed using the Niblack method.

**Results:**

While there was a significant difference between normal subjects and NTG patients for the peripapillary choroidal area (1,853,672 ± 626,501 μm^2^ vs. 1,606,448 ± 418,214 μm^2^, *P* = 0.047), there were no significant differences between the normal subjects and NTG patients observed for the macular choroidal area (345,365 ± 119,248 μm^2^ vs. 316,442 ± 85,732 μm^2^, *P* = 0.23). In the NTG patients, multivariate regression analysis demonstrated a correlation between the macular choroidal area and the axial length (β = -0.345, *P* = 0.04). Furthermore, there was also a significant correlation between the peripapillary choroidal area and the age of the NTG patients (β = -0.469, *P* = 0.004). In addition, there was no relationship between the glaucoma severity and the peripapillary and macular choroidal areas in the NTG patients.

**Conclusions:**

There was no correlation between the peripapillary choroidal area and glaucoma severity in NTG patients, even though the area was smaller in these patients.

## Introduction

The pathophysiology of glaucoma, which is a leading cause of blindness worldwide, has yet to be definitively established.[1] A loss of optic nerve fibers and associated visual field defects are characteristics of this neurodegenerative disease.[2] Since the main risk factor for the progression of glaucoma is an elevated intraocular pressure (IOP), the primary goal of glaucoma treatment is to try and control the IOP.[3] However, glaucoma progression can sometimes occur even when there is adequate IOP control. Non-IOP factors, such as vascular factors, are thought to have a greater role in normal-tension glaucoma (NTG) versus that found in primary open-angle glaucoma (POAG).

Since metabolic support for the prelaminar portion of the optic nerve head has been shown to be provided by the choroid [4–6], it has been speculated that the choroid may have an important role in glaucoma.[7–9] The development of the new methodology, enhanced depth imaging (EDI) spectral domain optical coherence tomography (OCT), has now made it possible to perform in vivo cross-sectional imaging of the choroid.[10] As a result, investigators can use this new methodology to determine the choroidal thickness in glaucoma patients.[11–19] However, some studies that have investigated the choroidal thickness found no differences between normal and glaucoma patients [11–14,17], while other studies demonstrated there was a significantly thinner choroidal thickness in glaucoma versus control eyes [15,16,18,19].

The choroidal thickness measurements obtained in the previous studies were performed at 1.7 mm superior, temporal, inferior, and nasal to the optic disc center and at 1- and 3-mm nasal, temporal, superior, and inferior to the fovea. To determine the choroidal measurements in our own recent study, we examined a macular choroidal area that was 1,500 μm wide and a peripapillary choroidal area that consisted of a 1.7-mm area around the optic nerve disc center.[20] By using an increased area for our measurements, this made it possible to collect a much greater amount of information from the choroid as compared to that reported for other previous studies.

The overall aim of this study was to examine the peripapillary and macular choroidal area measurements and then compare them between normal and NTG eyes.

## Materials and methods

### Subjects

Between July 2017 and March 2018, all eligible patients received a detailed explanation of the study at Kagawa University Hospital. In accordance with the principles outlined in the Declaration of Helsinki, all enrolled patients provided written informed consent. The study protocol was approved by the Kagawa University Faculty of Medicine Institutional Review Board.

Examinations performed in all of the study subjects included central corneal thickness (CCT), central and peripheral fields, gonioscopy, refraction, slit lamp, and visual acuity. Normal subjects, which included normal volunteers, underwent age- and axial length-matched measurements. Patients were required to have a spherical refraction within ± 6.0 diopters (D) and a cylinder within ± 2.0 D in order to be included within the study. This study excluded any subject who had any history of retinal diseases (e.g., diabetic retinopathy, macular degeneration, retinal detachment), and if they had poor image quality due to unstable fixation, severe cataract or if they had undergone previous laser therapy. Subjects with a previous treatment history with medications known to affect retinal thickness (intravitreal anti-VEGF therapy) were also excluded from the study. In all of the cases, EDI-OCT examinations were performed by the same investigator.

### EDI-OCT

The Heidelberg Spectralis (Heidelberg Engineering, Heidelberg, Germany) with the EDI-OCT technique was used to obtain the macular or peripapillary choroidal images, with all measurements performed between 1300–1500 hours. During the macular region scans, the image was obtained by using seven horizontal lines of 30 × 10° through the center of the fovea. For the peripapillary region, the scan utilized a 360°, 3.4 mm diameter circle that was centered on the optic disc. The subsequent analysis was performed using the best quality image that was chosen from at least three previous scans. The area found between the outer portion of the hyperreflective line that corresponded to the retinal pigment epithelium (RPE) and the inner surface of the sclera was defined as the choroidal thickness.

### Binarization of the choroid EDI-OCT images

**Fig 1:**
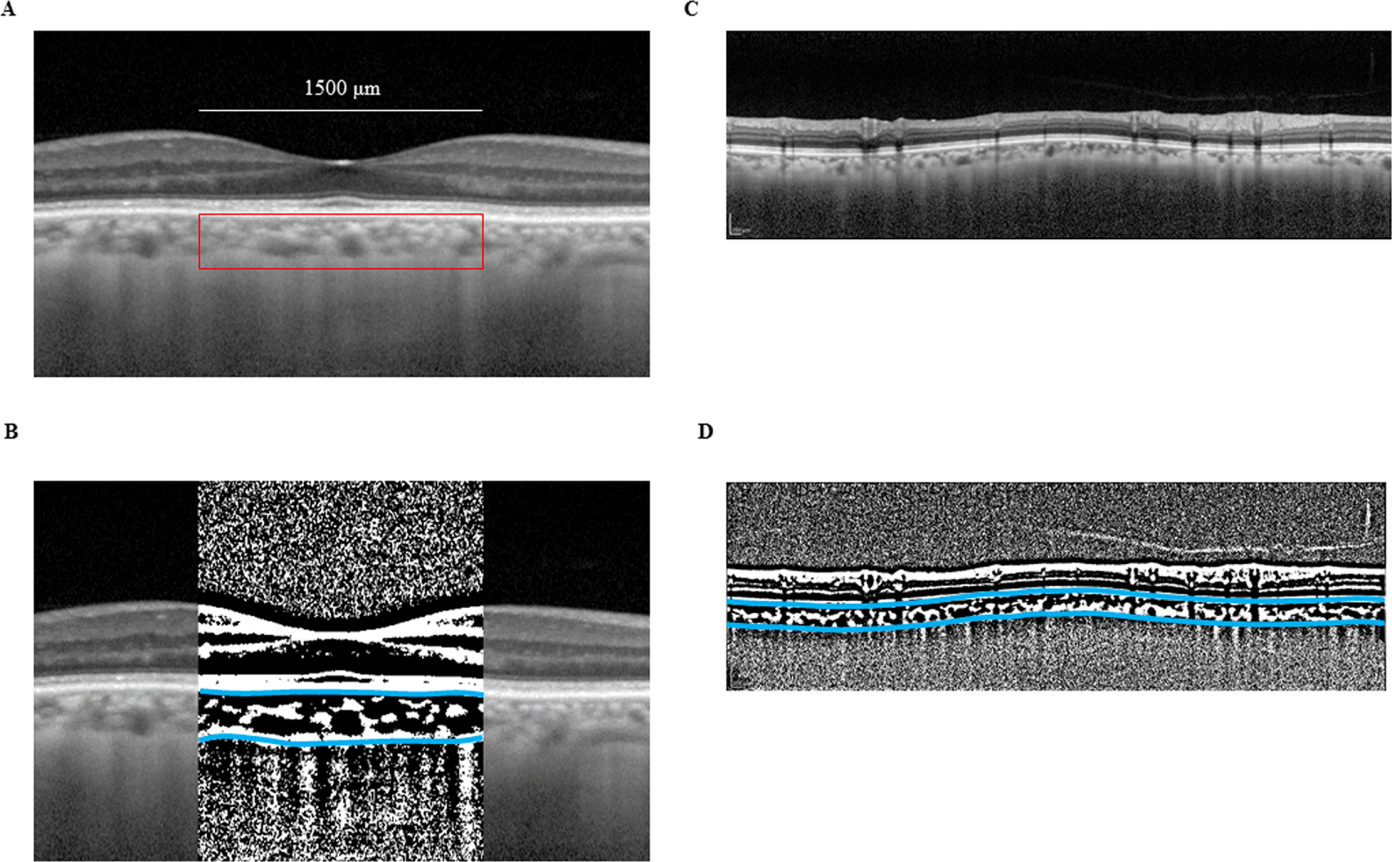
Enhanced depth imaging OCT image and converted binary image of the eye of a glaucoma patient. The EDI-OCT images in the macular area (A) or the peripapillary area (C) were converted to binary images (B, D) using ImageJ software. The luminal area (dark area) and the interstitial area are seen. The area between the blue lines indicates the measurement area of the choroid.

After recording and then masking the best of the EDI-OCT images, they were then displayed on a computer screen. Each of the images was evaluated by a single author (HK). The choroidal area in each of the EDI-OCT images underwent binarization using the previously described modified Niblack method.[20,21] Briefly, the EDI-OCT images were first analyzed using the ImageJ software (version 1.47, NIH, Bethesda, MD), with the analysis examining an area of the macular choroid that was 1,500 μm wide and which extended vertically (Fig 1A and 1B). The examined region included a 1.7-mm area around the optic nerve disc center (Fig 1C and 1D) that ranged from the retinal pigment epithelium to the chorioscleral border. All areas analyzed were determined by the ImageJ ROI Manager. After randomly selecting 3 choroidal vessels with lumens > 100 μm using the Oval Selection Tool on the ImageJ tool bar, we then averaged the reflectivities of these lumens. The average reflectivity was set as the minimum value, in order to reduce the noise in the OCT image. Subsequently, after the image was first converted and adjusted to 8 bits using the Niblack Auto Local Threshold program, the binarized image was once again converted to a RGB image. Conversions were required due to technical requirements for the binarization procedures and the automated calculations performed by the ImageJ program. To determine the hyporeflective area, the Threshold Tool was used, with dark pixels defined as hyporeflective areas, while light pixels were defined as hyperreflective areas. After adding data on the relationship between the distance on the fundus and the pitch of the pixels in the EDI-OCT images, which is dependent on the axial length, the hyperreflective and hyporeflective areas were then automatically calculated. The light pixels were defined as the interstitial choroid, and the dark pixels were defined as the luminal area.

### Statistical analysis

All statistical analyses were performed using SPSS for Windows (SPSS Inc., Chicago, IL). An independent Student’s *t*-test was used to analyze the differences for age, intraocular pressure (IOP), systolic blood pressure (SBP), diastolic blood pressure (DBP), ocular perfusion pressure (OPP), CCT, and axial length. OPP was defined as OPP = 2/3[DBP + 1/3(SBP–DBP)]–IOP. In both groups, the relationship between the choroidal area and various factors including age, axial length, CCT, IOP, DBP, OPP, and visual field mean deviation (MD) were analyzed by univariate and multivariate linear regression. Variables that showed a value of *P* < 0.2 in the univariate regression were included in the subsequent multivariate regression. The correlation between glaucoma severity (MD) and choroidal area was examined by using Spearman rank order correlations. *P* < 0.05 was considered statistically significant. All statistical values are presented as the mean ± standard deviation (SD).

## Results

Clinical characteristics of the enrolled subjects are presented in Table 1. Mean age in the normal subjects was 64.9 ± 11.9 years (range, 40–85 years) while it was 67.5 ± 11.5 years (range, 34–86 years) in the NTG patients (*P* = 0.41). No significant differences were observed for gender (*P* = 0.45), IOP (*P* = 0.94), axial length (*P* = 0.20), or OPP (*P* = 0.50).

**Table 1:** Clinical characteristics of normal subjects and normal-tension glaucoma patients.

	Normal	NTG	*P* value
Age (y)	64.9 ± 11.9	67.6 ± 11.5	0.41
Gender (M/F)	22/30	12/23	0.45
IOP (mm Hg)	14.9 ± 3.4	14.9 ± 3.7	0.94
Axial length (mm)	24.3 ± 1.7	24.7 ± 1.6	0.20
Central corneal thickness (μm)	526.2 ± 39.9	516.4 ± 29.7	0.23
Initial systemic blood pressure (mmHg)			
Systolic	127.1 ± 15.8	131.3 ± 20.7	0.30
Diastolic	75.3 ± 11.0	76.5 ± 15.4	0.67
Ocular perfusion pressure (mmHg)	46.8 ± 7.1	48.2 ± 12.2	0.50
MD range (dB)		-32.71 ~ -2.08	

NTG; normal-tension glaucoma, M; male, F; female, IOP; intraocular pressure

MD; mean deviation

Mean peripapillary choroidal areas were 1,853,672 ± 626,501 μm^2^ and 1,606,448 ± 418,214 μm^2^ in the normal subjects and NTG patients, respectively (*P* = 0.047) (Table 2). Although there was no significant difference between normal subjects and NTG patients for the luminal area (*P* = 0.16), there was a significantly smaller interstitial area in the NTG group versus the normal subjects (*P* = 0.002) (Table 2). Macular choroidal, luminal, and interstitial areas were 345,365 ± 119,248 μm^2^, 225,406 ± 84,357 μm^2^, and 119,959 ± 37,995 μm^2^ in normal subjects and 316,442 ± 85,732 μm^2^, 207,527 ± 61,829 μm^2^, and 108,915 ± 26,938 in NTG patients, respectively (Table 2). There were no significant differences found between normal subjects and NTG patients.

**Table 2:** Choroidal area observed in EDI-OCT images.

	Peripapillary choroidal area			Macular choroidal area		
	Normal	NTG	*P* value	Normal	NTG	*P* value
Total area (μm^2^)	1853672 ± 626501	1606448 ± 418214	0.047	345365 ± 119248	316442 ± 85732	0.23
Luminal area (μm^2^)	1041866 ± 445378	916751 ± 311181	0.16	225406 ± 84357	207527 ± 61829	0.29
Interstitial area (μm^2^)	811807 ± 190364	689697 ± 125360	0.002	119959 ± 37995	108915 ± 26938	0.15

NTG; normal-tension glaucoma

Univariate regression revealed there was a significant negative relationship with age for the peripapillary choroidal area of the NTG patients and normal subjects (NTG; β = -0.469, *P* = 0.004, normal; β = -0.477, *P* = 0.0004). Univariate regression additionally indicated there was a significant association with age (β = -0.278, *P* = 0.046) and CCT (β = 0.313, *P* = 0.042) for the macular choroidal area of the normal subjects. However, there was no association with each factor observed for the macular choroidal area of the NTG patients. Multivariate regression analysis demonstrated there was a significant relationship for age (β = -0.391, *P* = 0.004), axial length (β = -0.453, *P* = 0.001), and CCT (β = 0.283, *P* = 0.02) with the macular choroidal area of normal subjects. In addition, there was a significant relationship between the macular choroidal area and axial length (β = -0.345, *P* = 0.04) in the NTG patients. Tables 3 and 4 summarize the data on the relationships found between the choroidal area and the various factors. There was no correlation between glaucoma severity and peripapillary choroidal area (r = -0.61, *P* = 0.73) or macular choroidal area (r = 0.046, *P* = 0.80).

**Table 3:** Univariate regression analysis of choroidal area with associated factors.

	Peripapillay choroidal area				Macular choroidal area			
	Normal		NTG		Normal		NTG	
	β	*P* value	β	*P* value	β	*P* value	β	*P* value
Age	-0.477	0.0004	-0.469	0.004	-0.278	0.046	-0.101	0.56
Axial length	-0.036	0.80	-0.1	0.95	-0.272	0.051	-3.17	0.06
CCT	0.123	0.39	0.204	0.24	0.314	0.023	0.18	0.30
IOP	0.083	0.56	-0.175	0.32	0.059	0.68	0.79	0.65
OPP	-0.018	0.90	0.046	0.79	0.057	0.69	0.014	0.94
BP								
Systolic	-0.058	0.69	0.06	0.73	0.152	0.28	0.233	0.18
Diastolic	0.072	0.62	-0.051	0.77	0.013	0.93	-0.89	0.61
MD			-0.186	0.284			0.056	0.75

CCT; central corneal thickness, IOP; intraocular pressure, OPP; ocular perfusion pressure BP; blood pressure, MD; mean deviation

**Table 4:** Multivariate regression analysis of macular choroidal area with associated factors.

	Normal		NTG	
	β	*P* value	β	*P* value
Age	-0.391	0.004		
Axial length	-0.453	0.001	-0.345	0.04
CCT	0.283	0.02		
Systolic BP			0.269	0.11

CCT; central corneal thickness, BP; blood pressure

## Discussion

The present study was designed to examine the difference in the macular and peripapillary choroidal areas between NTG patients and normal subjects. Peripapillary choroidal areas were significantly decreased in NTG patients, even though the macular choroidal areas were similar between the normal subjects and NTG patients.

Hayreh et al.[22] examined the optic nerve head (ONH) blood supply and revealed that the branches of the short posterior ciliary artery and the circle of Zinn-Haller enter the peripapillary choroid. As a result, this ensures that blood will be supplied to the ONH. Based on these findings, it is our belief that measurements of the peripapillary choroidal area adjacent to the ONH can be very useful, as they reflect the ONH blood supply. Several other studies that examined the peripapillary choroidal thickness have reported finding that thickness was significantly reduced in glaucomatous versus healthy eyes.[15,19,23,24] In contrast, multiple other studies were unable to detect any association between the peripapillary choroidal thickness and glaucoma.[12,25,26] However, it should be noted that the authors of these other studies only focused on a few separate linear measurements. Therefore, in order to determine the choroidal measurements in our current study, we examined a macular choroidal area that not only was 1,500 μm wide, but also extended vertically to 1.7 mm around the optic nerve disc center. As compared to the other previous studies, the measurements that we made over this increased area made it possible for us to obtain a much larger amount of information from the choroid.

While there was a significant difference for the peripapillary choroidal area between the control subjects and NTG patients, we found that the peripapillary luminal area did not differ. Kinoshita et al.[27] examined the total choroidal area, CCT, and choroidal vessel luminal area in 38 healthy subjects and found that these appeared to fluctuate diurnally. However, they did not find any changes for the mean stromal area, which suggests that a change in the size of the large choroidal vessels was largely responsible for the diurnal variation that was observed in the choroidal thickness. Our recent study that examined changes after trabeculectomy found that while the reduction in the IOP led to an increase in the macular and peripapillary choroidal areas, this increase was primarily due to an increase in the luminal areas.[20] However, in our current study it remains unknown as to why we found no difference in the peripapillary luminal area even though there was a difference for the peripapillary choroidal area.

A lack of differences in the macular choroidal thickness between normal subjects and glaucoma patients has been reported in multiple OCT studies.[13,14,17,23] However, in many of these studies, the choroidal thickness was measured under the fovea, at 1 or 3 mm nasal from the fovea, 1 or 3 mm temporal from the fovea, 1 or 3 mm superior from the fovea, or 1 or 3 mm inferior from the fovea. In contrast, macular choroidal area measurements in our current study were performed using 30 × 10° horizontal lines that went through the center of the fovea. Even though our current study measured a larger area, our findings still support the results of the other previous studies.

Studies using OCT angiography reported regional microvasculature dropout in the parapapillary choroidal layer in the area with parapapillary atrophy (PPA) in glaucoma patients.[28,29] We could not deny the possibility that choroidal microvascular was included in the interstitial area in the current study. However, since the image was obtained by using 3.4 mm diameter circle that was centered on the optic disc, measurement area did not include the area with PPA in our glaucoma patients.

There were some limitations in our current study. First, there were only a small number of subjects examined in our study. Thus, a further study with a larger number of subjects will need to be undertaken in order to address this issue. Second, all identifications of Bruch’s membrane and the inner scleral border had to be manually performed, as there was no OCT software available for performing automated segmentation. Third, the cross-sectional design of our study made it difficult to determine whether the change of the peripapillary choroidal area preceded or followed the glaucomatous damage. Mundae et al.[30] recently examined the mean peripapillary choroidal thickness in glaucoma patients versus healthy control subjects at baseline. Their results showed that while glaucoma patients exhibited a significantly thinner thickness, the mean rate of the peripapillary choroidal thickness change was not significantly different between the healthy control subjects (follow-up period: 1.6 years) and glaucoma patients (follow-up period: 3.0 years). A longer prospective study will need to be performed in order to specifically determine the relationship between the peripapillary choroidal area and glaucoma progression.

## Conclusions

In conclusion, in NTG patients, while there were decreases in the peripapillary choroidal area in NTG patients, these changes were not influenced by the glaucoma severity. Moreover, a significant decrease was not observed in the macular choroidal area.
